# Antiviral Activity of Biocompatible Bionanocatalysts against Rotavirus

**DOI:** 10.1021/acsomega.4c10792

**Published:** 2025-06-18

**Authors:** Tessy Lopez-Goerne, Francisco J. Padilla-Godínez, Gabriela de la Rosa, José Manuel de la Rosa

**Affiliations:** † Department of Health Care, Autonomous Metropolitan University − Xochimilco, Mexico 04960, Mexico; ‡ Laboratory of Nanotechnology, 7180 National Institute of Neurology and Neurosurgery, Mexico 14269, Mexico; § Department of Mathematics and Physics, Western Institute of Technology and Higher Education, Tlaquepaque 45604, Mexico; ∥ Department of Development Genetics and Molecular Physiology, National Autonomous University of Mexico, Cuernavaca 62210, Mexico; ⊥ Graduate School Unit, Superior School of Mechanical and Electrical Engineering Zacatenco, Mexico 07738, Mexico

## Abstract

Rotavirus remains a significant cause of gastroenteritis, especially in infants and young children, leading to severe dehydration and even death in resource-limited settings. While vaccines are available, they offer incomplete protection, and effective antiviral treatments are lacking. This study investigates the antiviral potential of biocompatible bionanocatalysts against rotavirus, focusing on both pre- and postinfection stages. Such structures consist of nanostructured materials composed of pure or mixed oxides that form a heterogeneous catalyst with a full dispersion of the active metal, exhibiting potentiated catalytic properties (compared to common solid acid catalysts) and organic functional groups that mimic cellular ligands, thus endowing them with biocompatibility and affinity. Bionanocatalysts were synthesized and characterized, showing a uniform nanoscale distribution and high surface reactivity. Tests using the MA104 cell line demonstrated that these structures could significantly reduce viral infectivity when administered prior to viral exposure, likely by interacting with viral surface proteins and degrading the RNA genome. The treatment also reduced infectivity postinfection, though to a lesser extent. Importantly, the bionanocatalysts showed no cytotoxicity in uninfected cells, underscoring their safety and specificity. This research highlights the potential of bionanocatalysts as a novel antiviral therapy for rotavirus, providing a promising addition to existing preventive measures, particularly in areas with limited access to vaccines.

## Graphical abstract

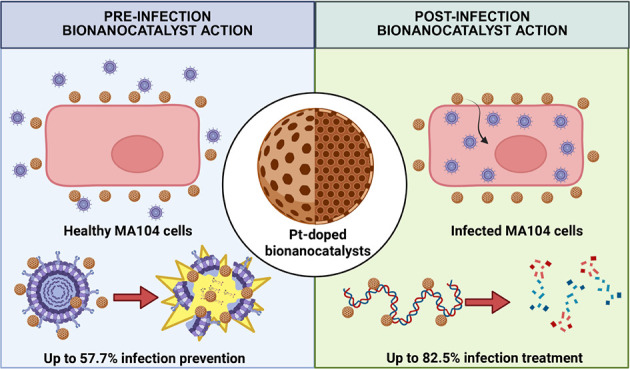

## Introduction

Rotavirus is a highly infectious pathogen responsible for severe gastroenteritis, primarily in infants and young children.^1,2^ The virus has a distinctive triple-layered protein shell that protects its double-stranded RNA genome, enabling it to withstand harsh environmental conditions.^3^ Rotavirus spreads mainly through the fecal-oral route,^4^ causing a rapid onset of symptoms, including profuse watery diarrhea, vomiting, fever, and dehydration.^5^ The virus specifically targets the epithelial cells of the small intestine, leading to cell death, disrupted nutrient absorption, and significant fluid loss.^6,7^ This disease remains a major global health burden, contributing to nearly 200,000 child deaths annually, with the highest toll in low- and middle-income countries.^8^

Treatment for rotavirus is largely supportive, focusing on managing dehydration through oral rehydration salts or intravenous fluids to restore fluid and electrolyte balance.^9^ Despite the availability of vaccines like Rotarix and RotaTeq, which have effectively reduced the incidence and severity of rotavirus infections, they do not offer complete protection.^10^ Additionally, vaccine distribution is limited in many resource-constrained settings, leaving many vulnerable populations at risk.^11^ No specific antiviral therapies currently exist for rotavirus, and its genetic diversity and ability to evade immune defenses further complicate efforts to develop comprehensive treatments.^12^ This lack of effective and accessible therapies highlights the need for novel approaches to combating rotavirus infection.

Recent advances in nanotechnology have introduced bionanocatalysts as a promising new strategy for antiviral intervention.^13^ As defined by Lopez-Goerne et al., bionanocatalysts consist of nanostructured materials composed of pure or mixed oxides that exhibit potentiated catalytic properties (concerning common solid acid catalysts) and organic functional groups that mimic cellular ligands, thus endowing them with biocompatibility and affinity: Bionanocatalysts are designed to degrade the genetic material of pathogens with high specificity and efficiency.^14^ The bionanocatalysts selectively reduce the three primary bond types found in the macromolecule’s nucleotides—carbon–carbon, carbon–nitrogen, and carbon–oxygen—when it comes into touch with the organism genetic material.^15^ Selective catalytic reaction, which include the burning of hydrocarbons, the reduction of nitrogen oxides into N2 and O2, and the oxidation of carbon monoxide derive in a harm to the genomic sequence, mostly manifested as punctual defects as cytosine deaminations, depurinations, and depyrimidinations.^16^ Through these mechanisms, bionanocatalysts have demonstrated antimicrobial activity against a range of microorganisms, including bacteria, fungi, and viruses such as the influenza virus.^17^ The application of bionanocatalysts in targeting rotavirus presents a novel approach that could directly disrupt the virus’s replication cycle by degrading its RNA genome, potentially offering a new avenue for antiviral therapy. This approach addresses current gaps in rotavirus treatment by providing a targeted mechanism that could be effective even in the face of the virus’s genetic variability.

In this study, we have evaluated the antiviral efficacy of bionanocatalysts against rotavirus at both preincubation and postincubation stages of infection. By assessing their ability to prevent viral attachment and replication, we explore how these nanomaterials can be optimized for therapeutic use. Our findings could pave the way for new treatments that not only complement existing vaccines but also provide an alternative strategy in regions where vaccines are less accessible. The exploration of bionanocatalysts in antiviral therapy represents an exciting frontier in the fight against rotavirus, offering hope for more effective and widely available interventions.

## Methodology

### Surface-Coated Bionanocatalysts Synthesis

Bionanocatalysts were synthesized according to a procedure detailed in a previous study.^18^ In brief, a solution of deionized water and acetone was prepared and maintained at room temperature under continuous stirring. Silica and titania precursors were subsequently added dropwise under constant stirring. Once all the precursors were fully incorporated, the mixture was stirred until gelation occurred. Platinum precursor was then added to this solution for coating the matrix. The resulting nanostructures were obtained after a further drying process required for surface strengthening of the coating.

### Structural and Morphological Characterization

The grain size, morphology, and texture of the bionanocatalysts were analyzed using scanning electron microscopy (SEM) on a JEOL JSM-6010LV microscope, which was equipped with an energy-dispersive spectroscopic (EDS) microanalysis system (OXFORD). Particle size was further determined using transmission electron microscopy (TEM) with a JEOL JEM-2100F, operating at a voltage of 120 kV. Images were captured with a CCD Mega Vision (III) camera. The bionanocatalysts were used in their untreated state for the electron microscopy studies, as outlined in previous research.^18^ Furthermore, the electronic state of the platinum coating was determined by X-ray Photoemission Spectroscopy (XPS). XPS analyses were conducted using a VG-Microtech Multilab 3000 spectrometer equipped with a hemispherical electron analyzer and a monochromatic Mg Kα (1253.6 eV) 300 W X-ray source. Prior to spectrum acquisition, the samples were placed in the analysis chamber and maintained until a residual pressure of approximately 4 × 10^– 9^ Torr was achieved. The spectra were recorded with a pass energy of 50 eV. Peak intensities were determined by integrating each peak after subtracting the S-shaped background and fitting the experimental curves using a combination of Lorentzian (30%) and Gaussian (70%) profiles. All binding energies (BE) were referenced to the C-1s line at 284.9 eV, ensuring binding energy values with an accuracy of ± 0.2 eV.

### Biocompatibility Evaluation on Healthy Cells

The MA104 cell line was purchased from the American Type Culture Collection (ATCC, Manassas, VA). Cells cultured following standardized protocols to ensure consistency and reproducibility.^19^ In brief, cells were grown in Dulbecco’s modified Eagles medium (DMEM) supplemented with heat-inactivated 10% fetal bovine serum (FBS, Invitrogen, Carlsbad, CA) and 100 U/ml penicillin-100 g/mL streptomycin (Invitrogen), at 37 °C in a CO_2_ incubator. For all experiments, MA104 cell cultures were prepared in 48-well plates at a density of 1.3 × 10 ^5^ cells per well. The cells were exposed to 20 different concentrations of bionanocatalysts in culture medium, prepared in triplicate, by serial dilution. Before their application, the bionanocatalysts were sterilized by autoclaving at 120 °C for 25 min. The treated cells were observed under an optical microscope at 1, 2, 4-, 8-, 13-, and 24 h post-treatment to monitor morphological changes and cellular responses. To assess cell viability, the cultures were stained with trypan blue at 24 and 48 h after treatment. Cell viability was determined by counting the number of viable (unstained) and nonviable (stained) cells under a microscope.

### Viral Infection Inhibition Test

Following previous methods, MA104 cells in 96-well plates were washed twice with phosphate-buffered saline (PBS).^20^ Then about 1,000 focus-forming units (FFUs) of a trypsin-activated cell lysate containing rotavirus RRV (kindly provided by Professor Susana López, Biotechnology Institute, National Autonomous University of Mexico, Cuernavaca, Morelos, Mexico) was adsorbed to the cells for 45 min at 4 °C. After the adsorption period, the virus inoculum was removed, the cells were washed once with PBS, MEM was added, and the infection was left to proceed for 14 h at 37 °C. Two experimental setups were performed: (i) incubation of cells with the virus before treatment with bionanocatalysts, and (ii) incubation of cells with the virus that had been previously treated with bionanocatalysts. For both experiments, bionanocatalysts were serially diluted in culture medium to concentrations of 1:2n+1 (1:1, 1:3, 1:7, 1:15, 1:31, 1:63, and 1:127), as previously reported.^21^ To prepare bionanocatalyst dilutions, suspensions were serially diluted in culture medium using dilution plates. The final columns of each plate contained only culture medium, serving as virus-only controls. The dilution process began by adding culture medium to all wells. An equal volume of bionanocatalyst suspension was then added to the wells in the first column, doubling the total volume in those wells. The contents of the first column were thoroughly mixed using a multichannel pipet, and a fixed volume was transferred to the wells of the second column. This process of resuspension and transfer was repeated sequentially across the columns until the final dilution was achieved. The activated rotavirus RRV was diluted in culture medium to achieve approximately 300 infecting foci per well in the control column. An equal volume of virus was added to each well in the dilution plate, corresponding to the bionanocatalyst dilutions. Culture medium was added to the remaining wells. The bionanocatalyst-virus mixtures were resuspended using a multichannel pipet to ensure contact between the bionanocatalysts and the virus, followed by incubation for 1 h at 37 °C. After incubation, the dilution plates were removed from the incubator and resuspended. Next, the dilutions from each well of the dilution plate were transferred to 96-well culture plates containing MA104 cells that had been prepared a few days earlier. The plates were incubated for 1 h to allow the virus to absorb into the cells. After incubation, the plates were emptied, washed with culture medium to remove any unabsorbed virus, and then incubated for an additional 14 to 16 h with fresh DMEM to maintain cell viability. Following the incubation period, the plates were emptied and stained using immunoperoxidase, a technique that allows for the visualization of viral proteins expressed in infected cells under a microscope. Finally, the number of virus-infected cells was counted to evaluate the effect of the bionanocatalysts on viral infection.

### Data Analysis

The data obtained were processed in Excel 2013 and OriginPro 9.0. All assays were performed in triplicate. To determine whether significant statistical differences existed both in the triplicate assays for each sample and between the samples, the data were analyzed by one-way ANOVA with 95% confidence interval (*p* < 0.05).

## Results and Discussion

### Physicochemical Properties of Bionanocatalysts

#### Atomic and Electronic Composition

The XPS spectra of the studied nanostructures were characterized by broad bands as a result of the overlapping of atoms in different oxidation states and/or coordination numbers. As is well-known, the M-O binding energy values are generally referenced to the residual carbon, which is always present in the samples. This carbon can come from different sources, including reactions occurring during the gelation process. This results in a complex shaped peak assigned to the 1s energy level of the carbon. Figure 1 shows the survey spectra of the sample (Figure 1a), as well as the high-resolution spectra for the orbital levels Platinum 4f (Figure 1b), Titanium 2p (Figure 1c), Carbon 1s (Figure 1d), and Oxygen 1s (Figure 1e). The spectra shown have no charge correction.

**Figure 1:**
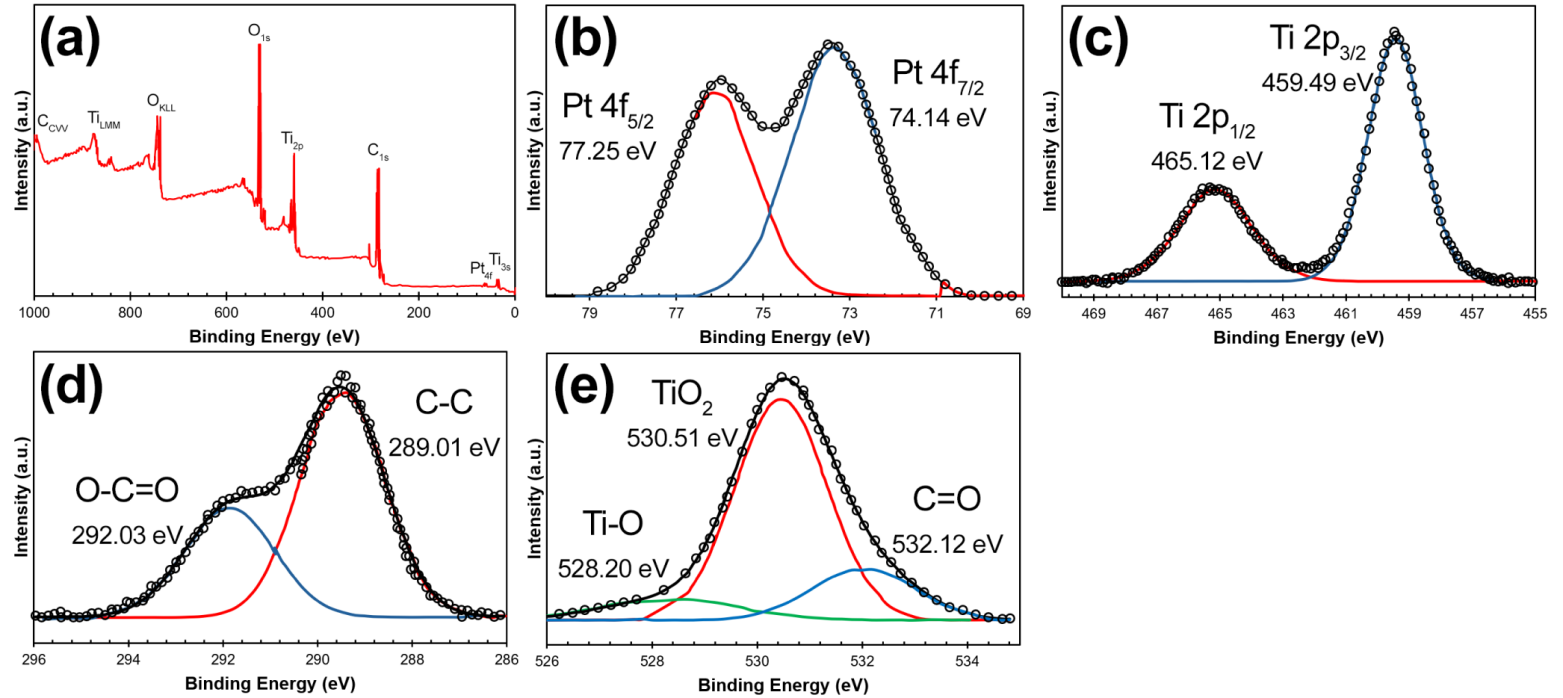
X-ray photoemission spectra of coated bionanocatalysts showing main elements: (a) survey, (b) Pt 4f, (c) Ti 2p, (d) C 1s, and (e) O 1s. All experiments were performed in triplicate.

The X-ray photoelectron spectroscopy (XPS) spectrum shown here is for the Pt 4f orbital, displaying two distinct peaks corresponding to the Pt 4f_7/2_ and Pt 4f_5/2_ spin–orbit components. The Pt 4f_7/2_ peak is located at a binding energy of 74.14 eV, while the Pt 4f_5/2_ peak appears at 77.25 eV. These peaks are characteristic of platinum and suggest the presence of metallic platinum or platinum oxides.^22^ The separation between the two peaks (approximately 3.1 eV) aligns with expected values for Pt 4f orbitals, confirming the oxidation state and chemical environment of platinum in the sample. The peak shapes are asymmetrical, which may indicate variations in the electronic environment, such as different oxidation states or interactions with other elements in the bionanocatalyst matrix. The intensity of the peaks, measured in arbitrary units (a.u.), reflects the relative abundance of each state, aiding in understanding the distribution of platinum in the synthesized nanostructures.

The spectra shown for the Ti 2p orbital indicates two main peaks, representing the Ti 2p_3/2_ and Ti 2p_1/2_ spin–orbit components.^23^ The Ti 2p_3/2_ peak is located at a binding energy of 459.49 eV, and the Ti 2p_1/2_ peak appears at 465.12 eV, with a spin–orbit splitting of approximately 5.6 eV. These binding energy values are consistent with titanium in the +4-oxidation state, which is characteristic of titanium dioxide (TiO_2_). The well-defined, symmetrical shape of these peaks suggests a stable and homogeneous chemical environment for titanium in the sample. Additionally, the lack of satellite peaks around these binding energies confirms the absence of other oxidation states, indicating the high purity of the TiO_2_ phase. The high binding energy for both peaks also reflects the presence of Ti^4+^ ions, further supporting that the Ti atoms are in an oxidized state consistent with TiO_2_ nanostructures. This spectral profile demonstrates the successful formation of TiO_2_ in the bionanocatalyst matrix.

In the C 1s region, at least 2 different carbon species separated by about 2 eV can be considered.^24^ If it is assumed that the high energy peak (HE= High energy) corresponds to residual carbon, the low energy peak (LE= Low energy) must correspond to carbonaceous species remaining from the sol–gel process. The intensity ratio (HE/LE) of these two peaks depends on the nature of the metal modifying the nanostructured particles. The highest value is for the pure oxide where its value is equal to 8.3 while the lowest is for the functionalized nanostructured particles, whose value is 0.5. Neither the separation energy nor the intensity ratios exhibit a trend with respect to the metal. These observations make it difficult to establish a precise scale for the spectra of all catalysts. By referencing the energy scale to the peak obtained by deconvolution of a broad band or a complex shaped band we increase the uncertainty in the energy scale, to avoid this uncertainty, the energy difference between the main peaks of Ti 2p and that of O 1s was obtained.

Figure 1E shows the spectrum for O 1s of the bionanocatalysts. The spectra can be described as the result of the deconvolution of three different species or groups of them. The first signal, at almost 528 eV is the broadest of the three and has a peak half-width maximum (fwhm) value of 2.5 ± 0.3 eV. The other two signals, show peaks at 530 and 532 eV respectively and fwhm values of 1.8 ± 0.1 eV. The peak signal centered at 530.1 ± 0.1 eV is in agreement with the values reported for titanium oxide. The last signal is attributed to the presence of hydroxyl groups, water or carbonates adsorbed on the surface of the material but also to organic molecules carrying oxygen atoms.^23^ This suggests that the presence of the peak at almost 530 eV corresponds to oxide ions in the nanostructured particles but also to other organic species, this may be due to the coordination of organic ligands, which may occur during the synthesis process. The low tethering energy value of the oxygen signal must be assigned to the oxygen bonded to the metal in polyhedral coordination. This is close to that found for different oxides. The surface concentration of the metals is quite low and the spectra have too much noise to be analyzed accurately.

#### Morphology and Particle Size

SEM images (Figure 2) depict bionanocatalyst clusters exhibiting varying morphologies and textural properties. The bionanocatalysts display a high degree of surface roughness across all images. In the top left image, the particles form irregular, loosely connected structures, indicative of a porous or agglomerated texture. In contrast, the top right image shows a denser aggregation of bionanocatalysts, suggesting stronger particle interactions and reduced porosity. Morphologically, the bionanocatalysts exhibit predominantly spherical to slightly elongated shapes. The lower left image offers a closer view of spherical particles, some of which appear fused, suggesting possible sintering during synthesis, resulting in larger aggregates. The bottom right image provides particle size measurements, showing a relatively uniform size distribution, with diameters ranging from 55.4 to 116 nm, with most particles between 60 and 100 nm. This consistent size distribution indicates controlled synthesis, with the small particle sizes being ideal for applications requiring high surface area, such as catalysis or drug delivery, where enhanced surface reactivity is beneficial.

**Figure 2:**
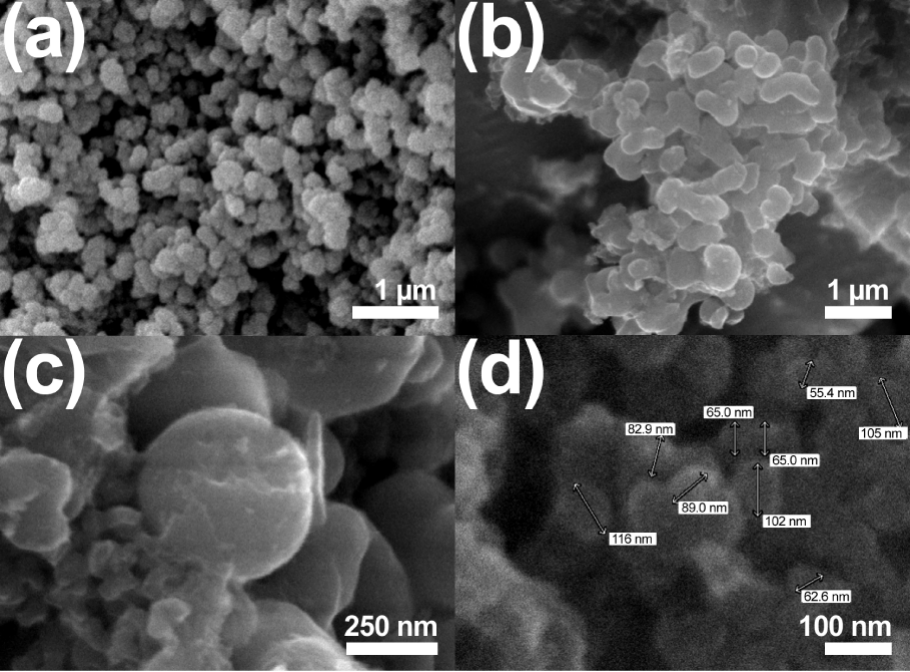
Scanning electron micrographs of coated bionanocatalysts at (a) 19,000×, (b) 30,000×, (c) 50,000×, and (d) 120,000×. All experiments were performed in triplicate.

Figure 3 shows the TEM micrographs providing detailed insights into the morphological and structural characteristics of the bionanocatalysts. Figure 3a shows a uniform distribution of the bionanocatalysts, with no significant aggregation, indicating a well-dispersed structure. The scale bar of 20 nm highlights the nanoscale size of the particles. Figure 3b further confirms the homogeneity of the nanostructures, showing a consistent distribution with a similar size range to the particles observed previously. Figure 3c displays larger aggregates with distinct dark regions, pointed out by the arrows, suggesting areas of denser material, likely indicating the formation of bionanocatalyst composite regions. Figure 3d returns to a view similar to the earlier figures, emphasizing the even distribution of the bionanocatalysts without the presence of significant clumping, ensuring that the composite remains at the nanoscale. Figure 3e presents a higher magnification (scale bar of 10 nm) view, focusing on the fine structural details of the bionanocatalysts. The image reveals an intricate network of interconnected particles, suggesting the formation of a mesoporous structure. Finally, Figure 3f shows a selected area electron diffraction (SAED) pattern, which displays concentric rings typical of polycrystalline materials, confirming the crystalline nature of the TiO_2_ phase within the nanostructures. The intensity and distribution of these rings indicate the presence of well-defined crystalline domains. These TEM studies confirm that the bionanocatalysts exhibit a well-dispersed, uniform morphology with crystalline features.

**Figure 3:**
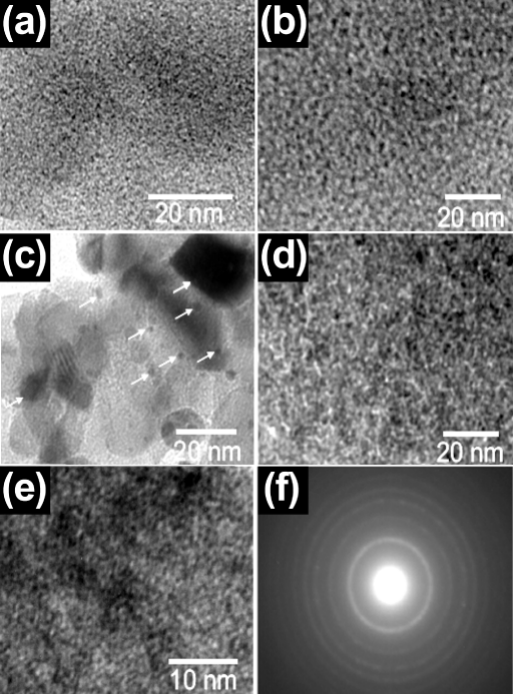
Transmission electron micrographs of coated bionanocatalysts at (a) 300,000×, (b) 400,000×, (c-–e) 500,000×, and (f) an electron diffraction pattern. A: Anatase; R: Rutile. All experiments were performed in triplicate.

### Biocompatibility Evaluation of Bionanocatalysts in Healthy Cells

Healthy, uninfected MA104 cells were exposed to the bionanocatalysts and observed under fluorescence inverted microscopy at various time points: 4 h (Figure 4a), 8 h (Figure 4b), 13 h (Figure 4c), and 24 h (Figure 4d) without any visible signs of cell damage. Treated cultures showed intact cell structures with clearly delineated membranes at 10× magnification. At 24 h (Figure 4e,g), trypan blue staining showed no dead cells at any concentration at 40x, and crystal violet staining revealed no morphological alterations in the monolayer, indicating a healthy cellular environment. At 48 h (Figure 4f,h), however, trypan blue staining indicated some cell death across all bionanocatalyst concentrations, though crystal violet staining still showed intact cell morphology, albeit with a slight reduction in cell numbers. This observation aligns with the expected cell death rate in monolayers incubated for this duration without a change in culture medium.

**Figure 4:**
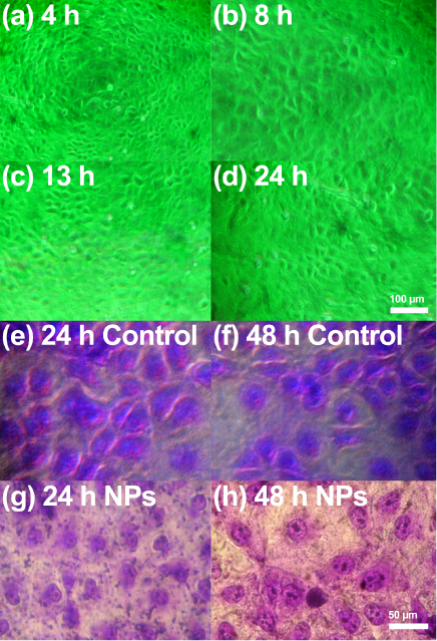
(a–d) Fluorescence inverted microscope images of MA104 cells exposed to bionanocatalysts at different times. (e–h) Crystal violet staining of cell cultures to evaluate qualitative cell viability. All experiments were performed in triplicate.

Photographic analysis of cells exposed to bionanocatalysts at 40× magnification after 24 or 48 h further confirmed the well-preserved cellular structure, even in the presence of bionanocatalyst agglomerates at higher concentrations. Notably, cells exposed to bionanocatalysts showed no significant cell death at 24 h, consistent with the known infectivity period of rotavirus (14 to 16 h), thus isolating the bionanocatalyst effects from viral activity. Future studies should corroborate quantitative cell viability at each concentration tested. Additionally, the exact concentration and size of bionanocatalyst clusters in suspensions remain uncertain, as sedimentation patterns vary with time. These clusters are handled as powders and dispersed in the culture medium via vortex agitation, forming turbulent solutions where sedimentation occurs over varying times. Suspensions are subsequently extracted for experimentation, though precise measurements of bionanocatalyst cluster characteristics are needed for accurate cell exposure analysis.

In previous studies, the biocompatibility of bionanocatalysts has been tested in different cell types and animal models to ensure viability and safety in their use.^25–28^ Although results have shown no side effects or immune responses associated with the use of bionanocatalysts, further research should be carried out with other cell lines, such as lymphocytes.

### Antiviral Activity of Bionanocatalysts Prior to Infection

To investigate the effect of bionanocatalysts on rotavirus infectivity, the virus was first activated with trypsin and incubated with bionanocatalysts for 1 h before being applied to healthy cells. Controls were implemented to compare the infectivity of the virus in the presence and absence of bionanocatalysts. Additional controls were included to evaluate cell viability when exposed to bionanocatalysts alone. Dilutions of bionanocatalyst suspensions were prepared in a stepwise manner, using culture medium across a dilution plate, with the final column reserved for virus-only controls. Each dilution was applied to MA104 cells, followed by the addition of the activated virus to infected wells.

After incubating the plates for 14 to 16 h with DMEM to maintain cell health, immunoperoxidase staining was performed to visualize viral protein expression in infected cells. The number of infected cells was then counted to assess the effect of bionanocatalysts on viral infectivity. Consistent with prior observations for other viral strains, a decrease in infectivity of at least 42.8% was noted when bionanocatalysts were applied prior to infection: notably, uninfected cells remained unaffected by bionanocatalyst exposure.^21^ For each bionanocatalyst concentration, the following infection percentages were observed: 1 pg/mL (43.7%), 10 pg/mL (57.2%), 100 pg/mL (56.6%), 1 ng/mL (57.0%), 10 ng/mL (45.7%), 100 ng/mL (54.2%), 1 μg/mL (48.8%), and 10 μg/mL (57.7%). Results showed a consistent decrease in viral infectivity with bionanocatalysts, and uninfected cells remained viable across all dilutions. The concentration of 10 ng/mL exhibited the greatest effectiveness in reducing infectivity, prompting further investigation into the most effective concentrations for enhanced antiviral activity.

### Antiviral Activity of Bionanocatalysts Posterior to Infection

In the postabsorption condition, where the virus were first absorbed by the cells before the application of bionanocatalysts, the following infection percentages were recorded: 1 pg/mL (79.2%), 10 pg/mL (82.5%), 100 pg/mL (76%), 1 ng/mL (76%), 10 ng/mL (70%), 100 ng/mL (71%), 1 μg/mL (79.2%), and 10 μg/mL (72.2%) (Figure 5). While the reduction in infection was less pronounced than in the preabsorption condition, the results still demonstrated a notable antiviral effect.

**Figure 5:**
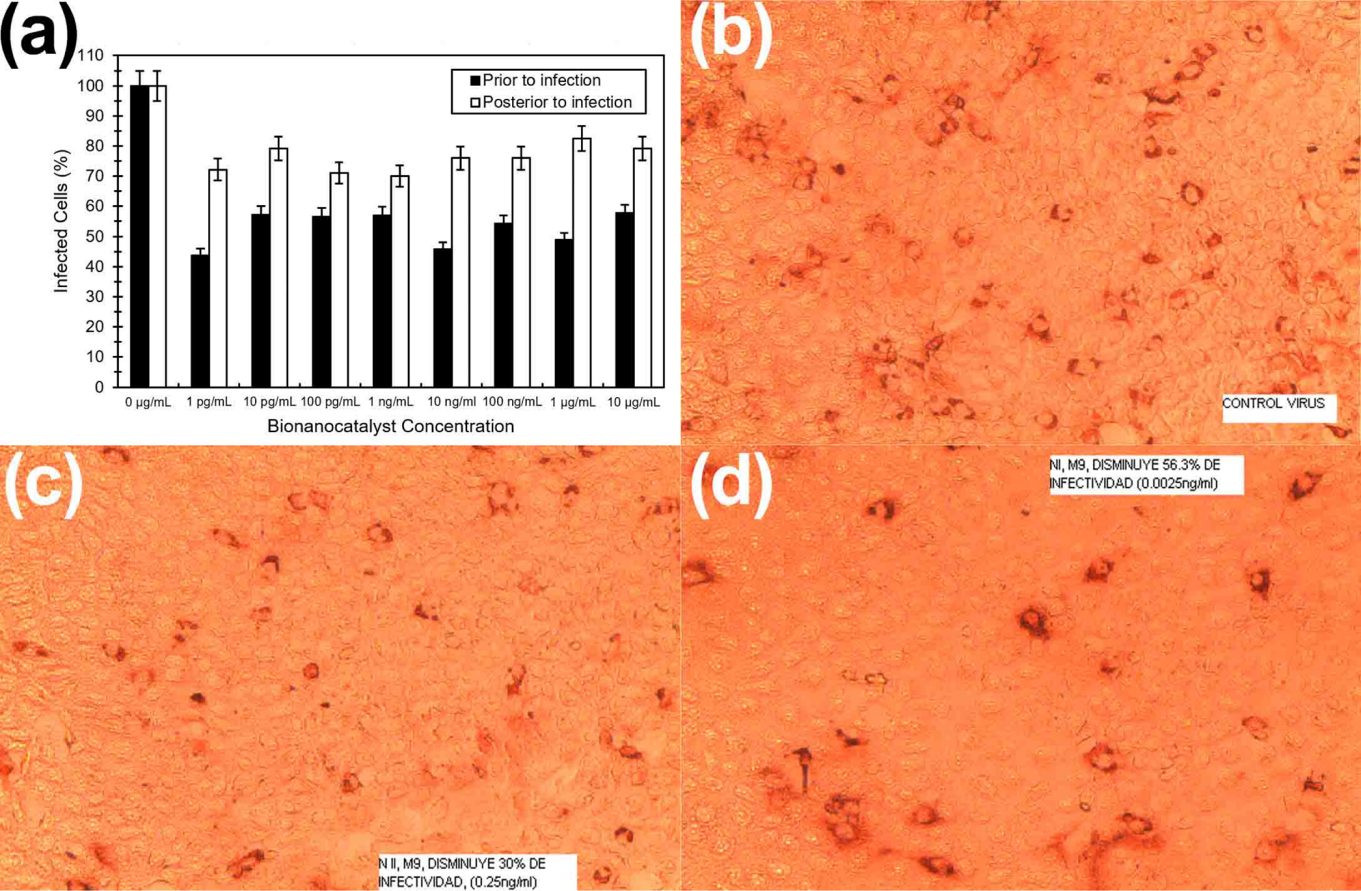
(a) Percentage of infected cells treated with bionanocatalysts prior and posterior to infection. (b–d) Micrographs showing cell morphology under infection: (b) control, (c) treated with bionanocatalysts posterior to infection, and (d) treated with bionanocatalysts prior to infection. All experiments were performed in triplicate: all data are expressed as the mean ± standard deviation.

Postabsorption tests showed a decrease in viral infectivity compared to the control, as visualized by immunohistochemical staining, which allowed for the precise counting of infected cells in each field. These findings underscore the specific action of bionanocatalysts on the virus, with a more significant effect observed when bionanocatalysts were applied before virus absorption.

It is important to note that the results in both pre- and postinfection studies show that there is no dose-related effect of the bionanocatalysts above 10 pg/mL (Figure 5a). This may be due to a saturation of the nanostructures on the extracellular matrix of MA104 cells, leading to a potenital accumulation and inability to internalize through the membranes. This could be solved by dissolving the bionanocatalysts in a biodispersing agent. Similarly, this additional step could increase the antiviral activity of the bionanocatalysts and achieve higher percentages of uninfected cells. Furthermore, given the closeness of the concentrations, no significant dose–effect is observed, although there seems to be a tendency. In this regard, we are currently working with more spaced concentrations (50 μg/mL) with higher concentrations so as to observe a more significant result. Although the current values are promising, further research is needed to enhance their activity.

Importantly, bionanocatalysts did not harm healthy cells, highlighting the specificity and low toxicity of the bionanocatalysts (Figure 5b–d). This specificity and safety profile suggest potential future pharmaceutical applications for bionanocatalyst in antiviral therapies.

## Conclusions and Outlook

The results of this study demonstrate the promising potential of bionanocatalysts as a novel antiviral therapy against rotavirus. Current treatment strategies for rotavirus are limited, primarily focusing on supportive care and prevention through vaccination, which is not universally accessible or fully effective. This study explores the application of bionanocatalysts, highlighting their ability to significantly reduce viral infectivity both before and after viral absorption into host cells. The findings offer an exciting alternative strategy to complement existing preventive measures, particularly in resource-limited settings where access to vaccines remains a challenge.

The physicochemical characterization of the synthesized bionanocatalysts confirmed their suitability for antiviral applications, with SEM and TEM analyses showing a well-dispersed, spherical morphology and uniform size distribution. The size range of 55 to 116 nm, combined with the high surface roughness, indicates a large surface area, which is critical for maximizing interaction with viral particles. These structural properties likely enhance the efficiency of viral inactivation, as demonstrated in both pre- and postinfection experimental setups.

The preabsorption condition, in which the virus was exposed to the bionanocatalysts before being introduced to healthy cells, showed a more significant reduction in rotavirus infectivity across a range of bionanocatalyst dilutions. This suggests that the bionanocatalysts are highly effective at disrupting the virus’s ability to attach to or enter the host cells, likely by interacting with viral surface proteins or directly degrading the viral RNA. The consistency of these results across multiple dilutions suggests the potential for these bionanocatalysts to serve as a prophylactic antiviral treatment. However, since such activity was tested only *in vitro*, further research is required both *in vivo* and in clinical trials before deciphering their final application.

In the postabsorption condition, where bionanocatalysts were applied after the virus had already been absorbed by the cells, the reduction in viral infectivity was less pronounced, though still significant. This indicates that while the bionanocatalysts can still exert their antiviral effects once the virus has entered the host cells, their primary mechanism of action may be more effective during the initial stages of infection. The fact that the bionanocatalysts did not harm healthy cells, as evidenced by trypan blue and crystal violet staining, further supports their potential as a safe therapeutic intervention.

One of the key advantages of bionanocatalysts is their specificity and low toxicity. The ability to selectively target rotavirus without inducing cytotoxic effects in healthy cells is a critical factor in developing antiviral therapies. This specificity, combined with the ability of the bionanocatalysts to maintain their antiviral activity across various dilutions and time points, underscores their robustness as a therapeutic option. Additionally, the study’s use of multiple analytical techniques, including light scattering, fluorescence, and refraction, ensured a thorough understanding of the bionanocatalyst characteristics, further validating their suitability for biomedical applications.

However, several questions remain regarding the precise mechanisms by which bionanocatalysts disrupt viral infectivity, particularly in the postabsorption stage. Future studies should focus on elucidating these mechanisms, potentially through more detailed molecular analyses of the virus-bionanocatalyst interactions. Additionally, optimizing the concentration and formulation of bionanocatalysts for maximum efficacy without compromising cell viability will be crucial as this technology moves toward clinical application.

In conclusion, the findings of this study highlight the potential of bionanocatalysts as a novel antiviral agent against rotavirus. Their ability to significantly reduce viral infectivity without damaging healthy cells, combined with their specificity and low toxicity, makes them a promising candidate for future pharmaceutical development. Further research is needed to optimize their use and explore their broader application in combating other viral pathogens. Nonetheless, this study represents an important step forward in the search for effective, accessible antiviral treatments, particularly in settings where current treatment options are limited.
